# First person – Eriko Seo

**DOI:** 10.1242/bio.041129

**Published:** 2019-01-15

## Abstract

First Person is a series of interviews with the first authors of a selection of papers published in Biology Open, helping early-career researchers promote themselves alongside their papers. Eriko Seo is first author on ‘Roles of Keber's valve and foot chamber for foot manipulation in the mussel *Nodularia douglasiae*’, published in BiO. Eriko is a postdoctoral fellow in the lab of Prof. Shigeaki Kojima at The University of Tokyo, investigating the physiology of living bivalves.


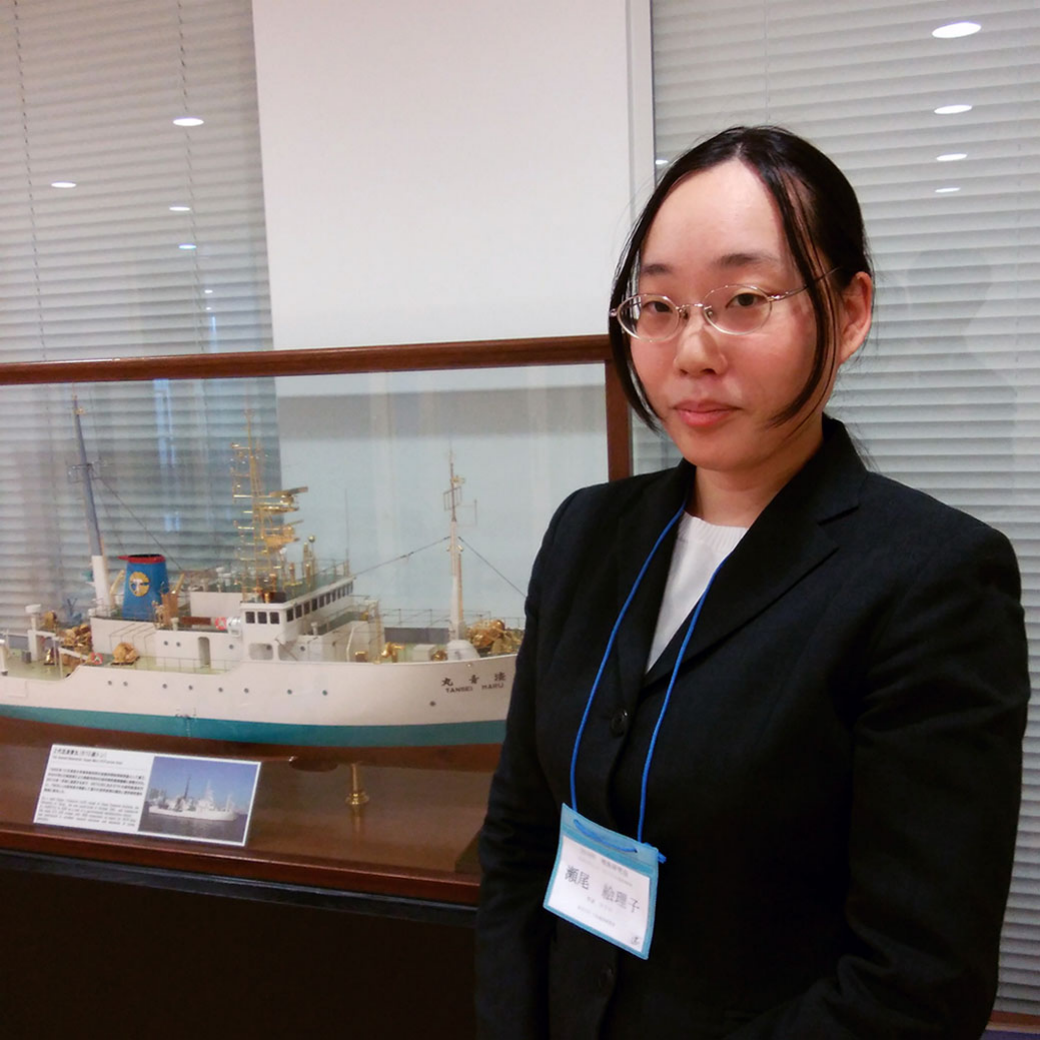


**Dr. Eriko Seo standing beside a model ship of the research vessel TANSEI MARU**

**What is your scientific background and the general focus of your lab?**

My subject is physiology of bivalves living in the deep sea. During my undergraduate course at Tokai University, I had the opportunity to study at JAMSTEC (Japan Agency for Marine-Earth Science and Technology), and investigated the reproduction and growth of clams in chemosynthetic biology communities as my Master's project. Then I moved to the laboratory of Prof. Shigeaki Kojima (Atmosphere and Ocean Research Institute, The University of Tokyo) as a PhD student. In my PhD project, I focused on non-invasive measurements of cardiac function of bivalves by using magnetic resonance imaging (MRI) and infrared photoplethysmogram. Now I work as a postdoctoral fellow, and collaborate with colleagues in the lab studying taxonomy, molecular phylogeny and ecology of benthic species (mussels and fishes) inhabiting the deep-sea.

**How would you explain the main findings of your paper to non-scientific family and friends?**

When we walk, our legs are extended or retracted by a combination of bone-joints and a pair of muscles. How do mussels move their feet? As mussels have no bones, they extend by filling their foot with hemolymph, and retract by contracting retractor muscles. However, the mussel has an open circulatory system. So they have to increase all the interstitial pressure, not only in the foot, when they extend foot. It seems to be a very inefficient system. In this study, we observed a fresh-water mussel: *Nodularia douglasiae* using an MRI (magnetic resonance imaging). We found that (1) the superficial muscle of its foot constitutes a closed space (the foot chamber) with an inlet (anterior aorta) and an outlet (Keber's valve). (2) The volume and internal pressure of the foot is controlled by opening and closing Keber's valve. Thus, any increase in pressure is limited inside the foot chamber, and the other organs are not affected by the high pressure. Therefore, this is a very efficient and safe system for controlling their feet. Mussels are more elegant organisms than we think.

“Mussels are more elegant organisms than we think.”

**What has surprised you the most while conducting your research?**

When I started this study I researched the literature on burrowing and Keber's valve in bivalves, and who had proposed the physiological process of their locomotion. However, I could not find any contemporary histological images of Keber's valve and only found sketches published more than 100 years ago! I am afraid that no one has been interested in Keber's valve for many years. The original article ­– Keber (1851) – proposed that the valve's function was as a lock preventing the venous from returning to the heart, and also suggested its contribution to the foot extension. Then, in the middle of 20th century, Trueman (1966) and Brand (1972) measured internal pressure in the clam and mussel foot, respectively, and linked Keber's valve to the foot movements in the digging cycle. But no one has tried to test the hypothesis of function of the Keber's valve thereafter. I was surprised that the idea of the digging cycle described in many text books has had no proof until now. In addition, I feel efforts to find the original article are important for reaching the final answer. I wish to acknowledge the support of the Bodleian Library and Google translate in German.

**What, in your opinion, are some of the greatest achievements in your field and how has this influenced your research?**

Ultrasound, X-ray and MRI are mainly developed and used in the medical field. The heartbeats of zebrafish were monitored by high resolution ultrasound imaging. Application of non-invasive techniques for invertebrates have also gradually increased in the last 10 years. However, bivalves have thick shells so it is difficult to observe soft tissues using X-ray, CT scans or ultrasound imaging. Therefore, I have set up an MRI system for bivalves. MRI could image not only anatomical structures, but also the motion of the heart and foot, direction and velocity of blood flow, kidney function, etc. These are distinctive merits in using MRI compared with other techniques.

**What's next for you?**

My next project is an alternative mechanism of foot extension and retraction in mussels that do not have Keber's valve. My preliminary results suggest the absence of Keber's valve in some mussels that live in the deep-sea. In addition, I would like to reveal the evolutionary processes behind the new mechanisms that were found.
